# Proteomic insights into dual-species biofilm formation of *E. coli* and *E. faecalis* on urinary catheters

**DOI:** 10.1038/s41598-024-81953-3

**Published:** 2025-01-30

**Authors:** Kidon Sung, Miseon Park, Ohgew Kweon, Angel Paredes, Alena Savenka, Saeed A Khan

**Affiliations:** 1 Division of Microbiology, National Center for Toxicological Research, Food and Drug Administration (FDA), Jefferson, AR, U.S.A.; 2 NCTR-ORA Nanotechnology Core Facility, FDA, Jefferson, AR, U.S.A.

**Keywords:** Dual-species biofilms, Urinary catheters, Proteome, Biofilms, Microbial communities

## Abstract

Infections associated with urinary catheters are often caused by biofilms composed of various bacterial species that form on the catheters’ surfaces. In this study, we investigated the intricate interplay between *Escherichia coli* and *Enterococcus faecalis* during biofilm formation on urinary catheter segments using a dual-species culture model. We analyzed biofilm formation and global proteomic profiles to understand how these bacteria interact and adapt within a shared environment. Our findings demonstrated dynamic population shifts within the biofilms, with *E. coli* initially thriving in the presence of *E. faecalis*, then declining during biofilm development. *E. faecalis* exhibited a rapid decrease in cell numbers after 48 h in both single- and dual-species biofilms. Interestingly, the composition of the dual-species biofilms was remarkably diverse, with some predominantly composed of *E. coli* or of *E. faecalis*; others showed a balanced ratio of both species. Notably, elongated *E. faecalis* cells were observed in dual-species biofilms, a novel finding in mixed-species biofilm cultures. Proteomic analysis revealed distinct adaptive strategies *E. coli* and *E. faecalis* employed within biofilms. *E. coli* exhibited a more proactive response, emphasizing motility, transcription, and protein synthesis for biofilm establishment; whereas *E. faecalis* displayed a more reserved strategy, potentially downregulating metabolic activity, transcription, and translation in response to cohabitation with *E. coli*. Both *E. coli* and *E. faecalis* displayed significant downregulation of virulence-associated proteins when coexisting in dual-species biofilms. By delving deeper into these dynamics, we can gain a more comprehensive understanding of challenging biofilm-associated infections, paving the way for novel strategies to combat them.

**Supplementary Information:**

The online version contains supplementary material available at 10.1038/s41598-024-81953-3.

## Introduction

Urinary tract infection (UTI) is a significant global health burden, with estimates suggesting 404.61 million cases and 236,790 deaths annually^1^. The extensive utilization of indwelling urinary catheters during hospital stays poses significant risks for patients, increasing susceptibility to associated complications^2^. Uropathogens have the capability to colonize the urinary tract and employ specific mechanisms to initiate infection, evade host defenses, and cause cellular damage^3^. Bacteria can infiltrate the bladder during catheter insertion, via the catheter lumen, or along the catheter-urethral interface^4^. Upon insertion, urinary catheters may impair the protective uroepithelial mucosa, thereby exposing new sites for bacterial adhesion^5^. Additionally, the presence of indwelling catheters in the urinary tract disrupts normal mechanical host defenses^6^.

Despite efforts to mitigate UTIs by using antimicrobial-coated catheters, some of these have been less efficacious in reducing infections^7,8^. Notably, catheters coated with silver had minimal impact on the formation of *Escherichia coli* and *Enterococcus faecalis* biofilms. Studies indicate that most urinary catheters harbor three or more bacterial species, including *E. coli* (28%), *Enterococcus* species (17%), *Pseudomonas aeruginosa* (14%), and *Klebsiella *species (8%)^9,10^. Yet, a smaller amount of research has examined interspecies interactions among UTI bacteria, particularly those forming polymicrobial biofilms on urinary catheters^11,12^. Competition between species within biofilms may amplify virulence potential and/or antimicrobial resistance^13^. In this study, using a global proteomic approach, we investigated a dual-species in vitro model to explore the effects of co-cultivating *E. coli* and *E. faecalis* on biofilm formation.

## Materials and methods

### Bacterial strains

This study employed two uropathogens, *E. coli* strain CFT073 and *E. faecalis* strain ATCC 29212. A single colony from overnight cultures on tryptic soy agar (TSA) plates (Hardy Diagnostics, Santa Maria, CA) was inoculated into separate 50-ml conical centrifuge tubes (Millipore Sigma, Burlington, MA) containing 5 ml of brain-heart infusion (BHI) broth (Hardy Diagnostics). Cultures were incubated overnight at 37 °C with shaking (200 rpm) using an Innova 4330 Refrigerated Incubator Shaker (New Brunswick Scientific, Edison, NJ). Bacterial cells were harvested by centrifugation at 20,817 x g for 10 min at 4 °C and washed three times with phosphate-buffered saline (PBS) (Thermo Fisher Scientific, Waltham, MA). The resulting pellets were resuspended in fresh culture broth and standardized for subsequent experiments.

### Biofilm formation on catheter segments

*E. coli* strain CFT073 and *E. faecalis *strain ATCC 29212 cultures were adjusted to 0.05 and 0.1 OD600 (optical density at 600 nm), respectively, in artificial urine media^14^. Cultures were added to separate wells of a 12-well tissue culture plate (Millipore Sigma) containing pre-cut (15 mm) anti-infective Foley catheters (BD, Franklin Lakes, NJ). The plates were incubated for 24 h at 37 ^o^C with shaking (130 rpm) in a refrigerated incubator shaker. After incubation, the planktonic cells were discarded. For the bacterial count, catheter segments were washed three times with PBS to remove loosely attached bacteria. Biofilms were detached by sonication (40 kHz, 5 min, Branson 5800 Ultrasonic Cleaner, Danbury, CT) and vigorous vortexing (5 min) before and after sonication^15^. The resulting bacterial suspension was serially diluted and plated on selective agar for enumeration. For the *E. coli* strain, we used MacConkey agar (Millipore Sigma) supplemented with 4 µg/ml vancomycin. For the *E. faecalis* strain, we supplemented Bile Esculin Azide Agar (Millipore Sigma) with 1 µg/ml gentamicin. Colony-forming units (CFU) were counted after overnight incubation at 37 ^o^C. All experiments were performed in triplicate, and data were presented as mean ± standard deviation. We used a two-way ANOVA to examine the effects of incubation time and culture condition on the CFU/cm^2^ of *E. coli* or *E. faecalis* in biofilms, along with their interaction. To identify specific differences between groups, we performed Tukey’s HSD post-hoc test, which showed that CFU/cm^2^ values were significantly higher in dual-species biofilms compared to single-species biofilms at all time points.

### Microscopic analysis of biofilms using Field Emission Scanning Electron Microscopy (FESEM)

Scanning Electron Microscopy (SEM) sample preparation was carried out as described^16^. Biofilm-grown catheter segments for 72 h were washed with PBS to remove loosely attached cells. Afterward, the samples underwent dehydration through a graded ethanol series (15%, 30%, 50%, 70%, 80%, 90%, 95%, and 100%). To further enhance drying and improve sample conductivity, we then treated them with hexamethyldisilazane (HMDS, Millipore Sigma) in a stepwise manner, including pure HMDS and mixtures of HMDS with ethanol (1:2, 1:1, and 2:1). Finally, the dehydrated samples were sputter-coated with gold (Denton Vacuum, Moorestown, NJ) for FESEM imaging. We employed a Zeiss-Merlin FESEM (Carl Zeiss Microscopy, Thornwood, NY) to acquire high-resolution images of the biofilms.

### Protein extraction

Proteins were extracted using a protocol adapted from the previously described method, with minor modifications^17^. Planktonic cells were grown in artificial urine media at 37 ^o^C for 15 h with shaking at 200 rpm. Biofilm cells were grown on catheter segments at 37 ^o^C for 72 h with shaking at 130 rpm. The washed planktonic and biofilm pellets were resuspended in 500 µl of BugBuster Plus Lysonase Kit (Millipore Sigma). This suspension was placed in Lysing Matrix B tubes (MP Biomedicals, Santa Ana, CA) containing 0.1 mm silica spheres. To disrupt the bacterial cells, we used an FP120 reciprocator (MP Biomedicals) at speed 6 for 45 s. Subsequently, the cells were subjected to boiling and vortexing for 5 min and 1 min, respectively. The final protein extract was obtained by centrifuging the suspension at 20,817 x g for 20 min at 4 ^o^C.

### Protein sample preparation and liquid chromatography with tandem mass spectrometry (LC-MS-MS) analysis

Extracted proteins were subjected to analysis for protein identification using mass spectrometry at MS Bioworks (Ann Arbor, MI). Briefly, the protein samples were digested with trypsin overnight. This involved a reduction step with 12 mM dithiothreitol and an alkylation step with 15 mM iodoacetamide, both performed at room temperature for 1 h each. Trypsin was added in a 1:20 enzyme-to-substrate ratio. The samples were then acidified with 0.3% trifluoroacetic acid and purified using a µHLB OASIS C18 desalting plate (Waters, Milford, MA).

For LC-MS/MS analysis, 1 µg of the protein pool was loaded onto a Waters M-class high performance liquid chromatography system interfaced with a ThermoFisher Exploris 480 mass spectrometer. Peptides were separated on a two-column system: a trapping column followed by a 75 μm analytical column. Both columns were packed with XSelect CSH C18 resin (Waters) with particle sizes of 5 μm for the trapping column and 2.4 μm for the analytical column. The column temperature was maintained at 55 °C. A 30-min gradient was used for elution. The mass spectrometer was operated in data-independent acquisition (DIA) mode.

Six sets of gas-phase fractionated precursor ions were acquired across a mass range of 396–1002 m/z. Each set involved a high-resolution full MS scan followed by a series of targeted MS/MS scans on 26 precursor ions with a 4 m/z isolation window. The product ion scans were staggered by 2 m/z to achieve comprehensive coverage. All scans were acquired at high resolution, specifically at 30,000 full width at half-maximum (FWHM) resolution. Automatic gain control (AGC) was set to 1e6 for both MS and MS/MS scans. The maximum ion injection time was set to 50 ms for full MS and dynamically adjusted for product ion scans, requiring at least nine data points across the peak. Normalized collision energy (NCE) was set to 30.

Scaffold DIA software 3.2.1 analyzed the raw mass spectrometry data (RAW files). This software converted the data to a standardized format (mzML) and deconvolved the staggered window acquisitions. It then aligned peptides based on chromatography retention times and searched them against a protein database and a custom reference library. To ensure high confidence, Scaffold DIA filtered the results using Percolator software (1% false discovery rate). Finally, the software quantified protein abundance by calculating peak areas for identified peptides (using the top 5 fragment ions) and normalized the data based on total protein intensity per sample. Protein identification was performed using a customized database comprising the trypsin-digested proteomes of *E. coli* CFT073 and *E. faecalis* ATCC 29212. The peptide and protein false discovery rates (FDR) were set at 0.01. *E. coli* and *E. faecalis *proteins were distinguished based on strain-specific peptide sequences that were unique to each organism in the proteomic database. Differentially expressed proteins (DEPs) were determined by filtering the proteomic data using a fold change threshold of ≥ 2.0, based on normalized protein intensity values. Protein function classification was carried out using the Cluster of Orthologous Groups (COG) system, and COG functional enrichment analysis was conducted on the identified DEPs^18^. Figures and statistical analyses were generated using a custom Python script.

## Results

### Biofilm formation of dual-species cultures on antimicrobial urinary catheter segments in artificial urine media

**Fig. 1:**
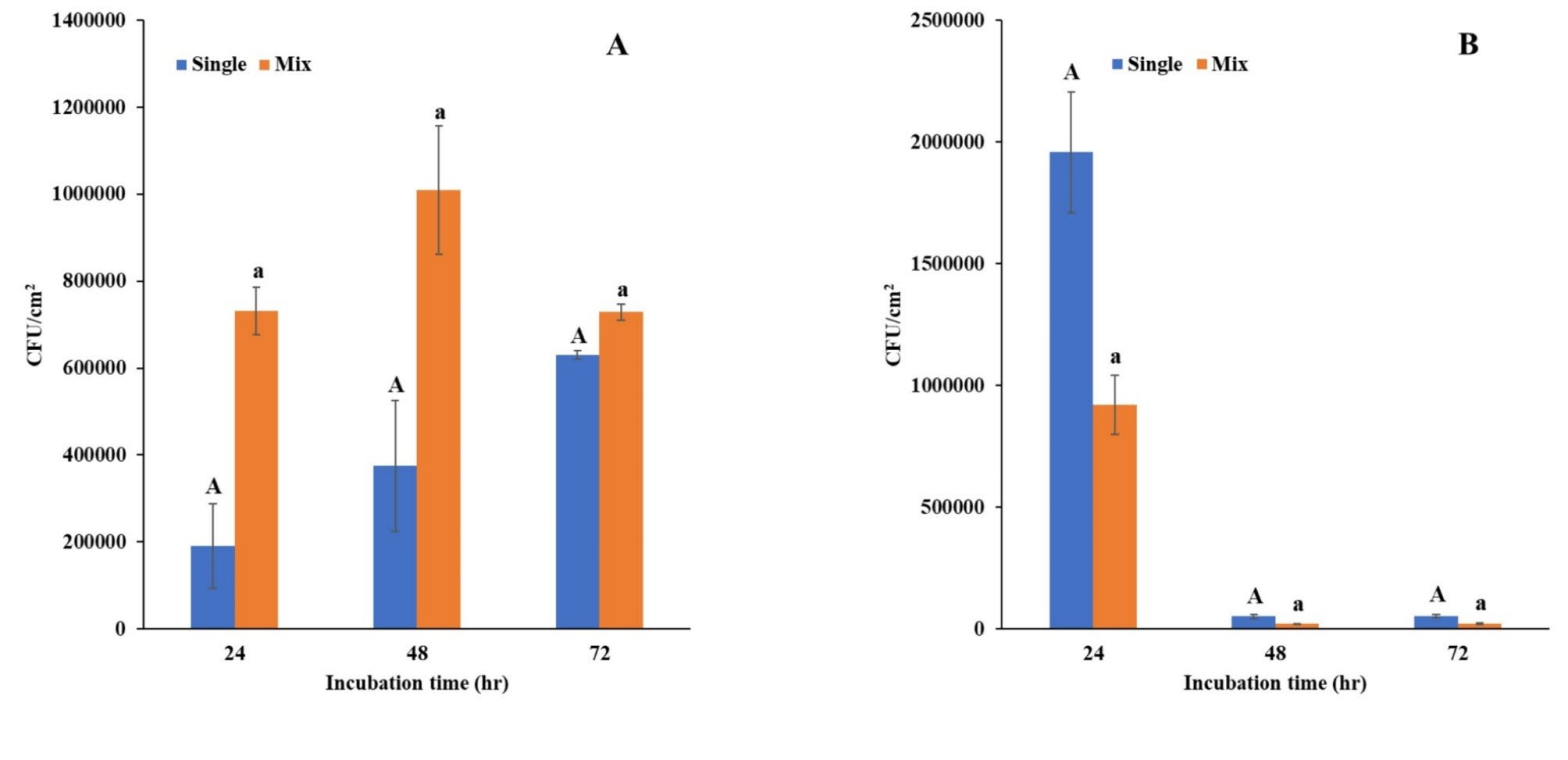
Biofilm formation in single- and dual-species models. This figure shows the average CFU/cm^2^ of *E. coli* CFT073 (**A**) and *E. faecalis* ATCC 29212 (**B**) in biofilms grown on urinary catheter segments in artificial urine media under single-species and dual-species culture conditions. Error bars represent the mean ± standard deviation. Different letters between the bars (e.g., “A” and “a”) indicate statistically significant differences between conditions (*p* < 0.05).

This study investigated biofilm formation by *E. coli* CFT073 and *E. faecalis* ATCC 29212 on antimicrobial urinary catheter segments in artificial urine media using a dual-species culture model (Fig. 1). To distinguish between *E. coli* and *E. faecalis *in the dual-species biofilm experiments, we determined the antibiotic susceptibility of each strain using the broth microdilution method^19^. *E. coli* was intrinsically resistant to vancomycin and susceptible to gentamicin (MIC 1 µg/ml), whereas *E. faecalis* exhibited resistance to gentamicin but susceptibility to vancomycin (MIC 4 µg/ml). To facilitate species differentiation, we used selective agar media. MacConkey agar, which exclusively supports the growth of gram-negative bacterial species like *E. coli*, was supplemented with 4 µg/ml vancomycin to inhibit *E. faecalis* growth. Conversely, Bile Esculin Azide agar, utilized for identifying enterococci, was supplemented with 1 µg/ml gentamicin to suppress *E. coli*. This approach enabled for the specific enumeration of each bacterial species within the dual-species biofilms.

Compared to single-species cultures, dual-species biofilms exhibited a significantly higher bacterial count in *E. coli*. Notably, *E. coli* in single cultures displayed a steady rise in CFU up to 72 h. In contrast, *E. coli* within dual-species cultures peaked at 48 h and declined by 72 h. *E. faecalis* showed an opposing trend. Single-species cultures had higher CFUs than did dual-species cultures at 24 h. Interestingly, both cultures exhibited a rapid decline in CFU by 48 h, reaching similar levels at 72 h.

### Microscopic analysis of biofilms

**Fig. 2:**
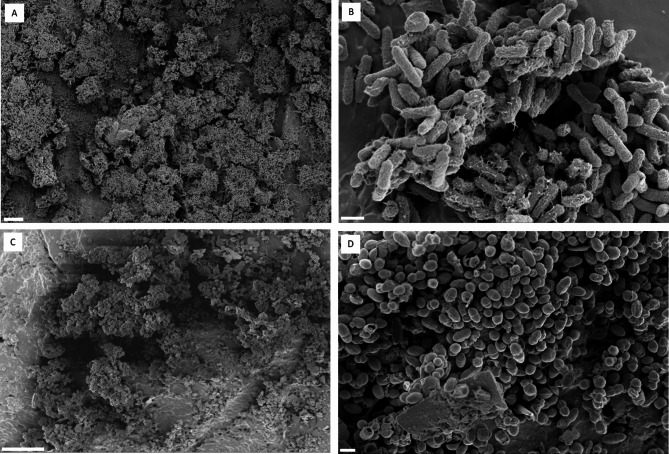
FESEM images of single-species biofilms formed by *E. coli* CFT073 and *E. faecalis* ATCC 29212 on antimicrobial urinary catheter segments. (**A)** and (**B)** depict *E. coli* CFT073 biofilm, while (**C)** and (**D)** show *E. faecalis* ATCC 29212 biofilm. (**A)** and (**C)** present lower-magnification views (scale bar = 10 μm) for visualizing overall biofilm structure. **(B)** and (**D)** offer higher-magnification views (scale bar = 1 μm) for detailed observation of bacterial morphology within the biofilm.

FESEM imaging of the *E. coli* biofilms on the antimicrobial catheter segment revealed dense, multilayered bacterial cells adhering to each other (Fig. 2A-B). This dense arrangement forms a thick, well-established three-dimensional biofilm structure. The biofilm cells produced copious amounts of extracellular polymeric substance (EPS), forming a continuous network that embeds and connects adjacent bacteria. Additionally, the cells exhibited production of flexible, fiber-like structures resembling pili on their surfaces. In contrast, *E. faecalis* biofilms, while dense and three-dimensional (Fig. 2C-D), showed less prominent EPS and pili compared to *E. coli.*

**Fig. 3:**
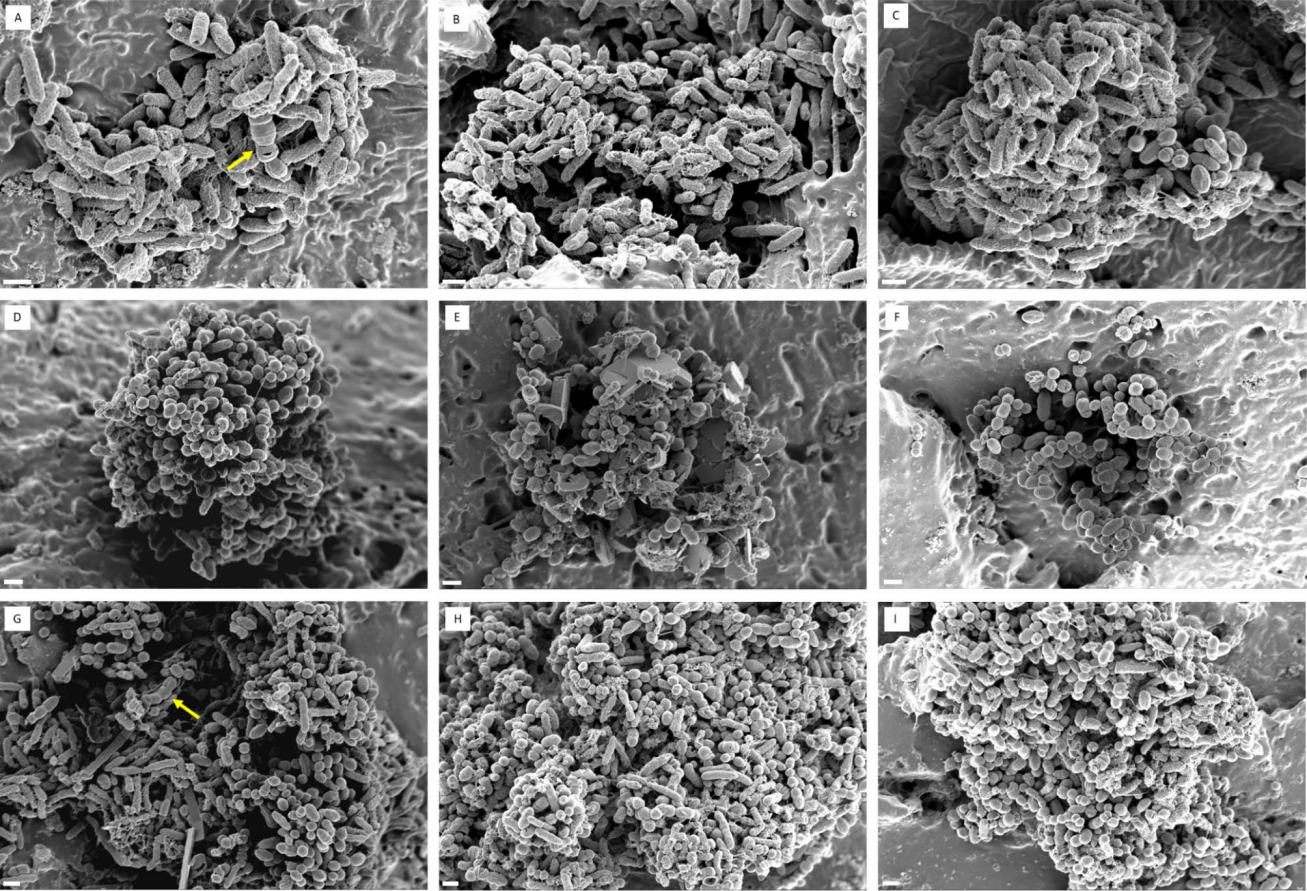
FESEM images of dual-species biofilms on urinary catheter segments. All panels utilize a scale bar of 1 μm. (**A-C**) Biofilms predominantly composed of *E. coli* CFT073. (**D-E**) Biofilms predominantly composed of *E. faecalis* ATCC 29212. (**G-I**) Biofilms containing a comparable ratio of *E. coli* CFT073 and *E. faecalis* ATCC 29212. Yellow arrows indicate elongated cells of *E. faecalis* ATCC 29212.

**Fig. 4:**
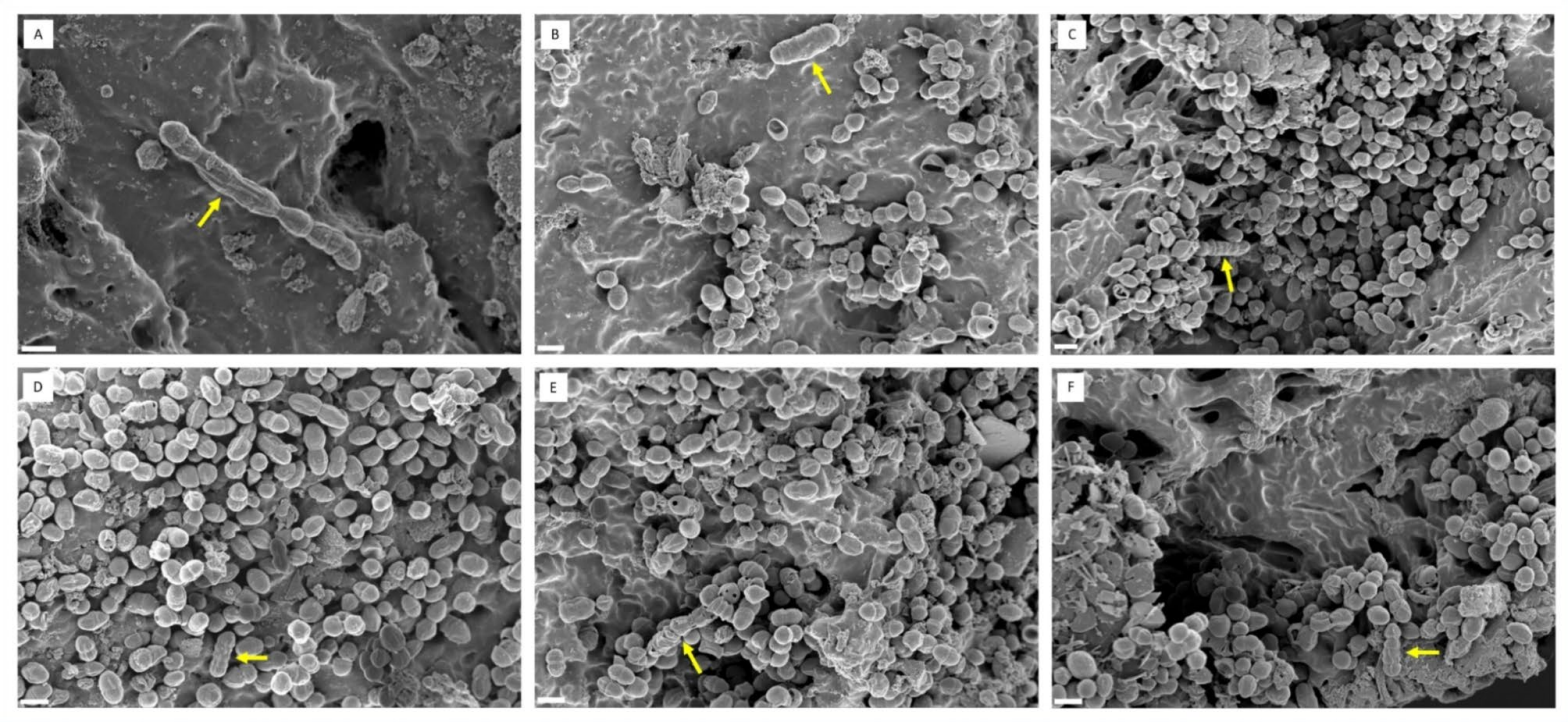
FESEM images of *E. faecalis* ATCC 29212 biofilms with elongated cells on urinary catheter segments. All panels use a scale bar of 1 μm. Elongated cells are indicated by yellow arrows.

Dual-species biofilms containing both *E. coli* and *E. faecalis* displayed varied compositions (Fig. 3). Some biofilms were dominated by *E. coli* (Fig. 3A-C), while others exhibited a higher prevalence of *E. faecalis* (Fig. 3D-F). Interestingly, some biofilms showed a more balanced ratio of both species (Fig. 3G-I). Notably, FESEM analysis also revealed elongated cells of *E. faecalis* within the biofilms (Fig. 4).

### Proteome expression profile

#### Identification of differentially expressed proteins from *E. coli* CFT073 biofilms

**Fig. 5:**
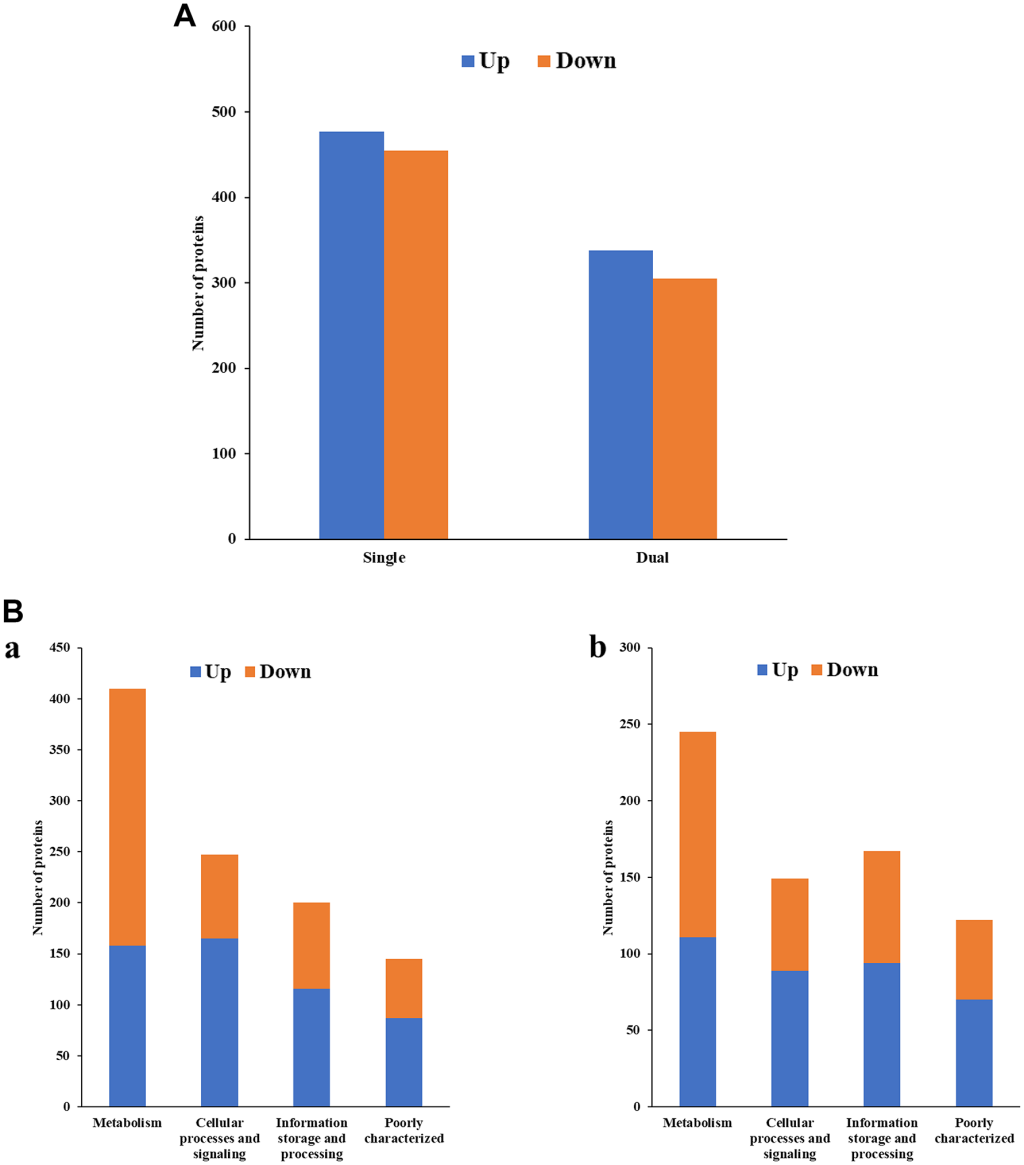

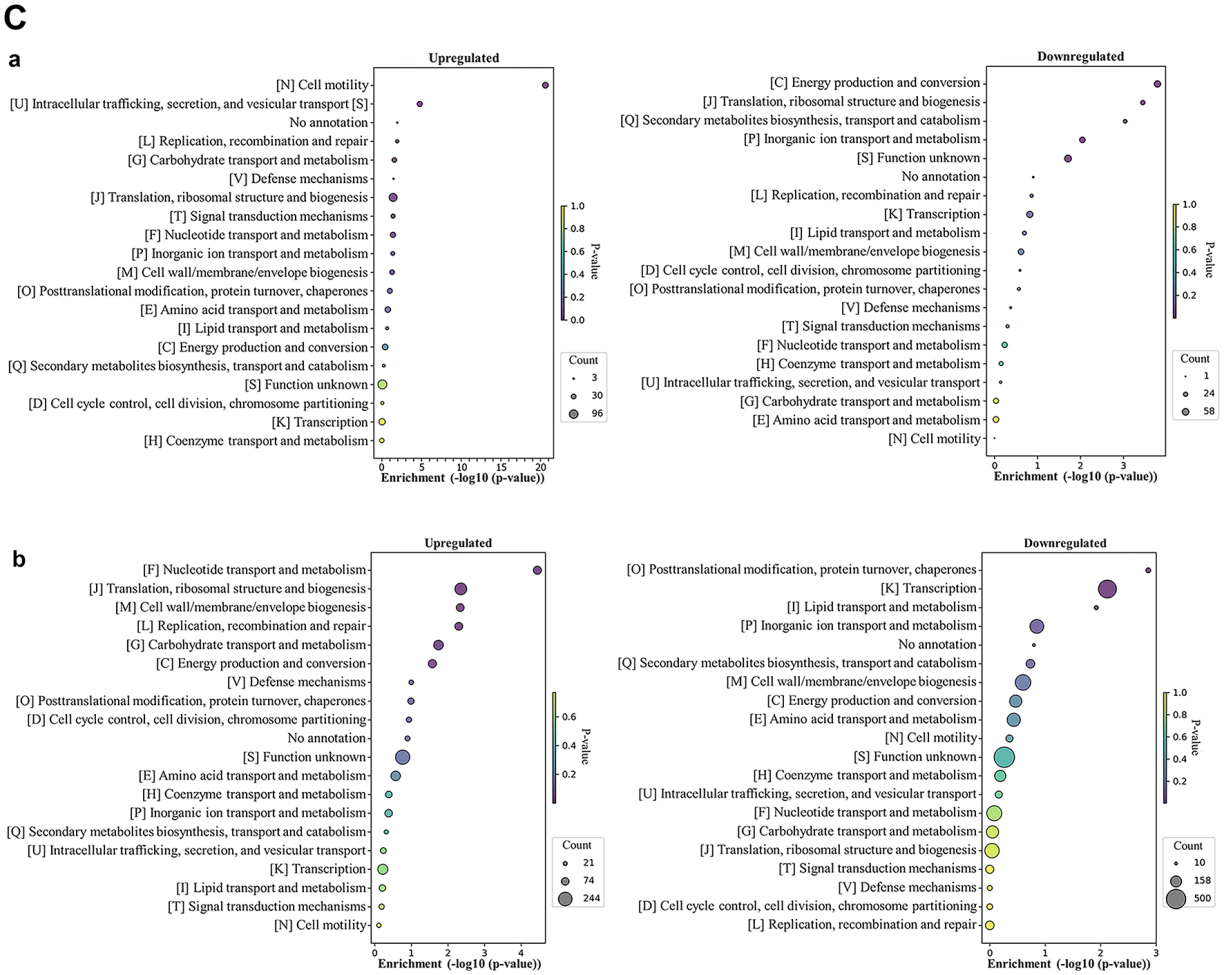
(**A**) Differentially expressed proteins identified from *E. coli* CFT073 biofilms (single- vs. dual-species), (**B**) COG functional classification of four major categories in differentially expressed proteins identified from *E. coli* CFT073 biofilms. a: Single-species, b: Dual-species, (**C**) COG functional enrichment analysis of differentially expressed proteins in *E. coli* CFT073 biofilms. a: Single-species, b: dual-species.

*E. coli* in single-species biofilms exhibited 477 upregulated and 455 downregulated proteins (Fig. 5A, Table S1). However, in dual-species biofilms, the numbers shifted to 338 upregulated and 305 downregulated proteins. According to COG functional category analysis, the proteins were classified into four main functional categories: Cellular processes and signaling, Information storage and processing, Metabolism, and Poorly characterized functions^18^. In both single- and dual-species biofilms of *E. coli*, a higher proportion of proteins in each COG category, except Metabolism, were upregulated compared to downregulated proteins (Fig. 5B). Notably, the Cellular processes and signaling category showed the most substantial difference in the number of upregulated proteins between single- and dual-species biofilms.

The detailed COG functional enrichment of differentially expressed proteins in single-species *E. coli* biofilms revealed several notable findings (Fig. 5C-a). Translation, ribosomal structure and biogenesis (J) had 61 upregulated proteins, the highest number. The Cell motility category (N) exhibited a remarkable upregulation, with 42 proteins showing increased expression compared to only 1 protein with decreased expression. Conversely, the Cell wall/membrane/envelope biogenesis category (M) showed a substantial 39 proteins downregulated. Interestingly, regulation of gene expression (Transcription category, K) appeared balanced, with a similar number of proteins upregulated (42) and downregulated (46). Categories related to metabolism (Energy production and conversion (C), Amino acid transport and metabolism (E), and Nucleotide transport and metabolism (F)) displayed a mix of upregulated and downregulated proteins.

The presence of *E. faecalis* in dual-species biofilms significantly impacted *E. coli*’s protein expression profile (Fig. 5C-b). While Cell motility (N) still showed upregulation (22 proteins), the level was considerably lower compared to single-species biofilms. Interestingly, Translation, ribosomal structure and biogenesis (J) remained upregulated (35 proteins). Like single-species biofilms, Cell wall/membrane/envelope biogenesis (M) exhibited substantial downregulation (29 proteins down vs. 11 up) in dual-species biofilms. Transcription (K) displayed extensive regulation with 44 upregulated and 38 downregulated proteins. Notably, Intracellular trafficking, secretion, and vesicular transport (U) showed significant upregulation (20 proteins) compared to downregulation (6 proteins). Finally, categories such as Energy production and conversion (C) and Inorganic ion transport and metabolism (P) exhibited a balance between upregulated and downregulated proteins, while Nucleotide transport and metabolism (F) showed a downregulation trend.

#### Identification of differentially expressed proteins from *E. faecalis* ATCC 29212 biofilms

**Fig. 6:**
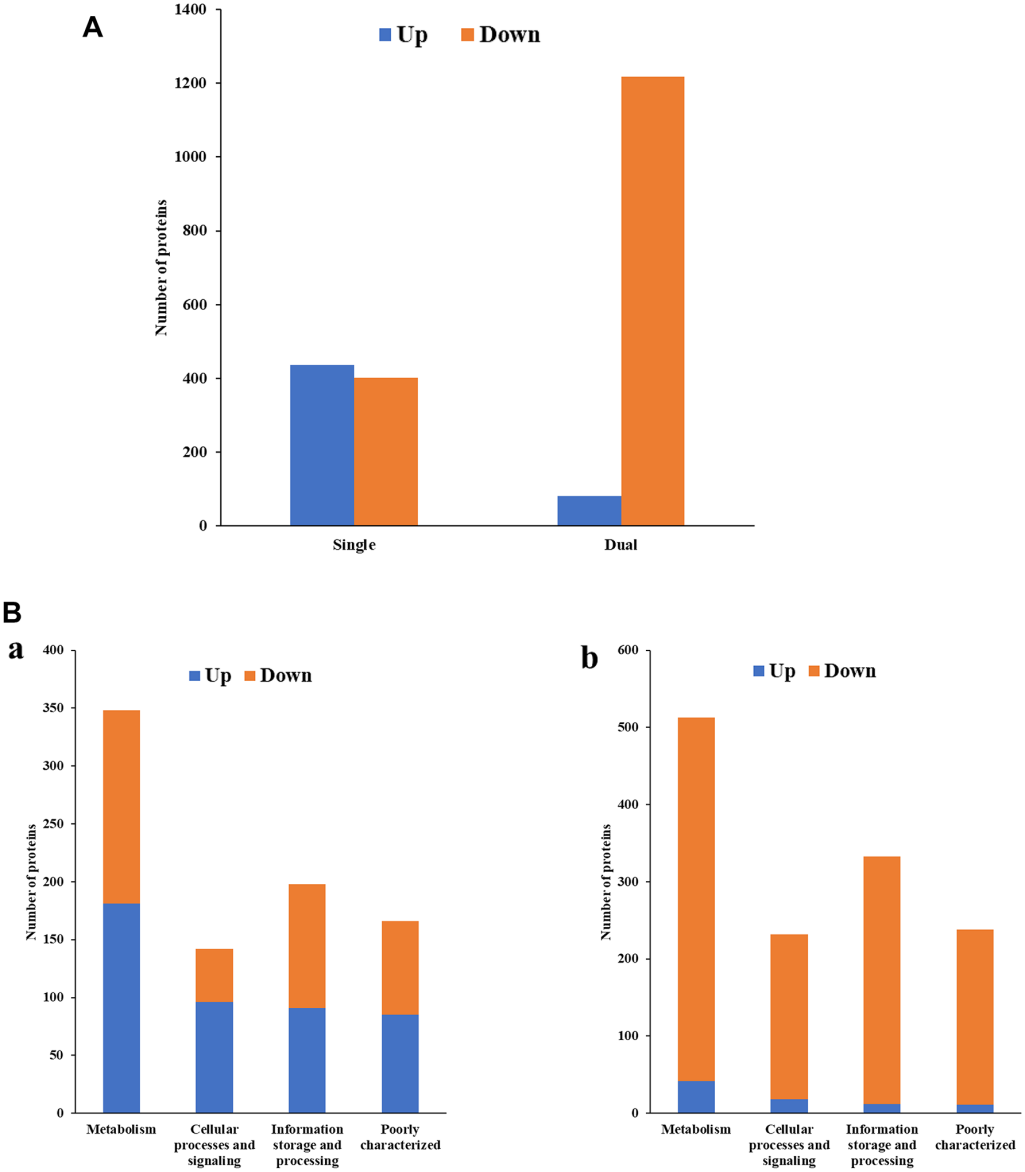

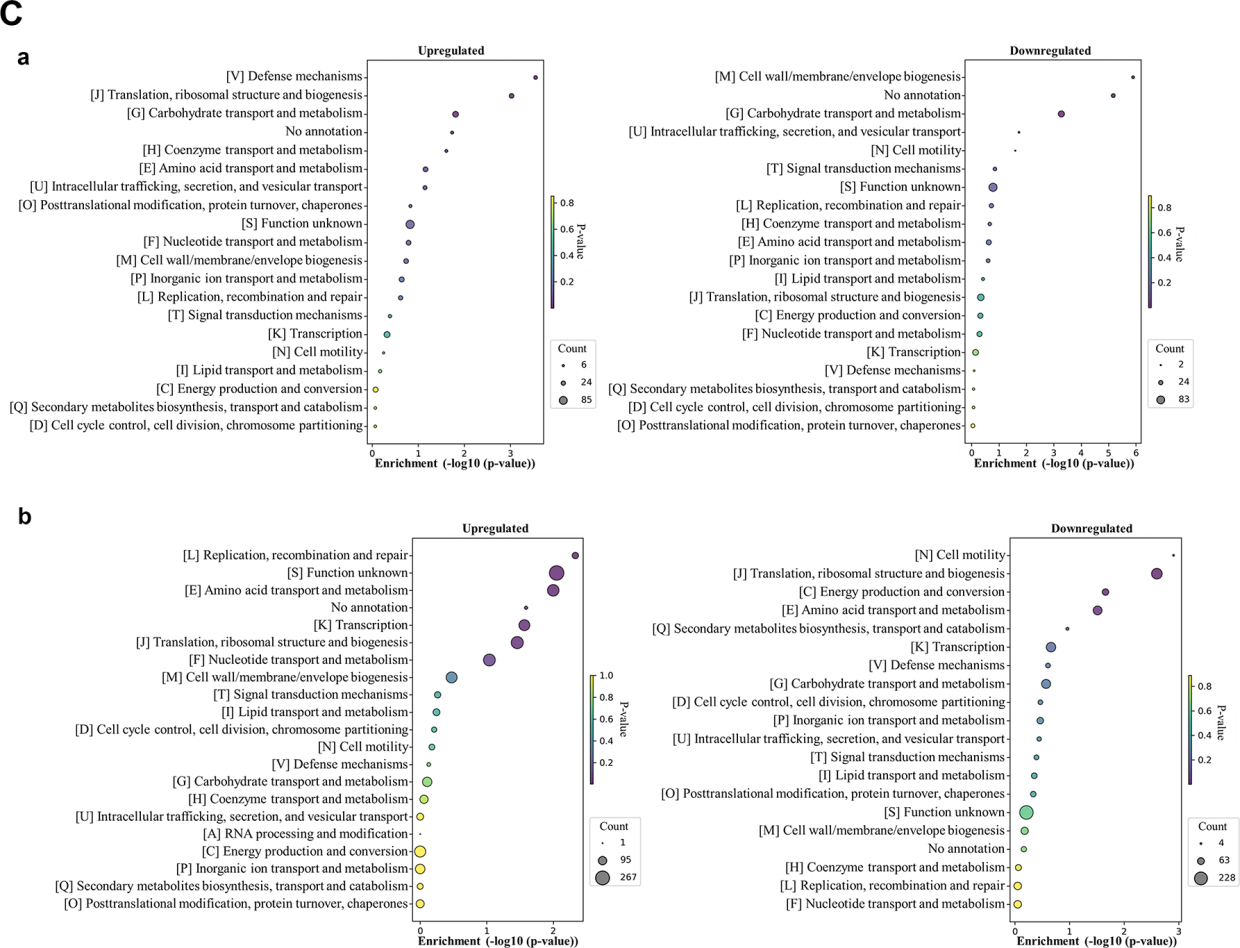
**(A)** Differentially expressed proteins identified from *E. faecalis* ATCC 29212 biofilms (single- vs. dual-species), **(B)** COG functional classification of four major categories in differentially expressed proteins identified from *E. faecalis* ATCC 29212 biofilms. **a**: Single-species, **b**: Dual-species, **(C)** COG functional enrichment analysis of differentially expressed proteins in *E. faecalis* ATCC 29212 biofilms. **a**: Single-species, **b**: dual-species.

When grown as a single-species biofilm, *E. faecalis* exhibited a modest increase in upregulated (436) compared to downregulated proteins (402) (Fig. 6A, Table S1). However, the trend dramatically reversed when *E. faecalis* was grown in a dual-species biofilm with *E. coli*, with a significant reduction in upregulated proteins (81) and a substantial increase in downregulated proteins (1,217) compared to the single-species biofilms.

In *E. faecalis* single-species biofilms, we observed a predominance of upregulated proteins in the Metabolism category (Fig. 6B). However, a striking shift occurred in dual-species biofilms. All COG categories, except for Information storage and processing, displayed a significant downregulation vs. upregulation of proteins. Notably, the Metabolism category showed the most prominent repression.

A detailed analysis of protein function (COG classification) revealed distinct patterns in *E. faecalis* biofilms, highlighting potential adaptations based on the presence or absence of *E. coli*. In single-species biofilms, *E. faecalis* displayed extensive regulation in the Transcription (K) category. This category showed 44 proteins upregulated and 37 downregulated (Fig. 6C-a). The Cell wall/membrane/envelope biogenesis (M) category exhibited substantial upregulation, with 27 proteins upregulated. Energy production in single-species biofilms appeared balanced. The Energy production and conversion category (C) had a similar number of proteins upregulated^30^and downregulated^23^. Translation, ribosomal structure, and biogenesis (J) presented a more nuanced picture, with a higher number of proteins downregulated (49) compared to those upregulated^24^. The Carbohydrate transport and metabolism category (G) exhibited extensive regulation, with a relatively even distribution of upregulated (40) and downregulated (47) proteins. Similarly, Amino acid transport and metabolism (E) displayed balanced regulation with significant numbers of proteins upregulated^27^and downregulated^27^.

Compared to single-species biofilms, dual-species biofilms showed a significant shift towards downregulation across various protein categories (Fig. 6C-b). The most dramatic change occurred in Translation, ribosomal structure, and biogenesis (J) with a substantial decrease (141 proteins downregulated) compared to only four proteins upregulated. Similarly, Transcription (K) exhibited a strong downregulation trend, with 112 proteins downregulated and four upregulated. Categories related to transport processes, Amino acid transport and metabolism (E) and Nucleotide transport and metabolism (F), also displayed a dominant downregulation pattern. The Carbohydrate transport and metabolism (G) category was highly regulated, showing 11 upregulated and 101 downregulated proteins.

#### Differentially expressed proteins associated with virulence in *E. coli* CFT073 biofilms

**Fig. 7:**
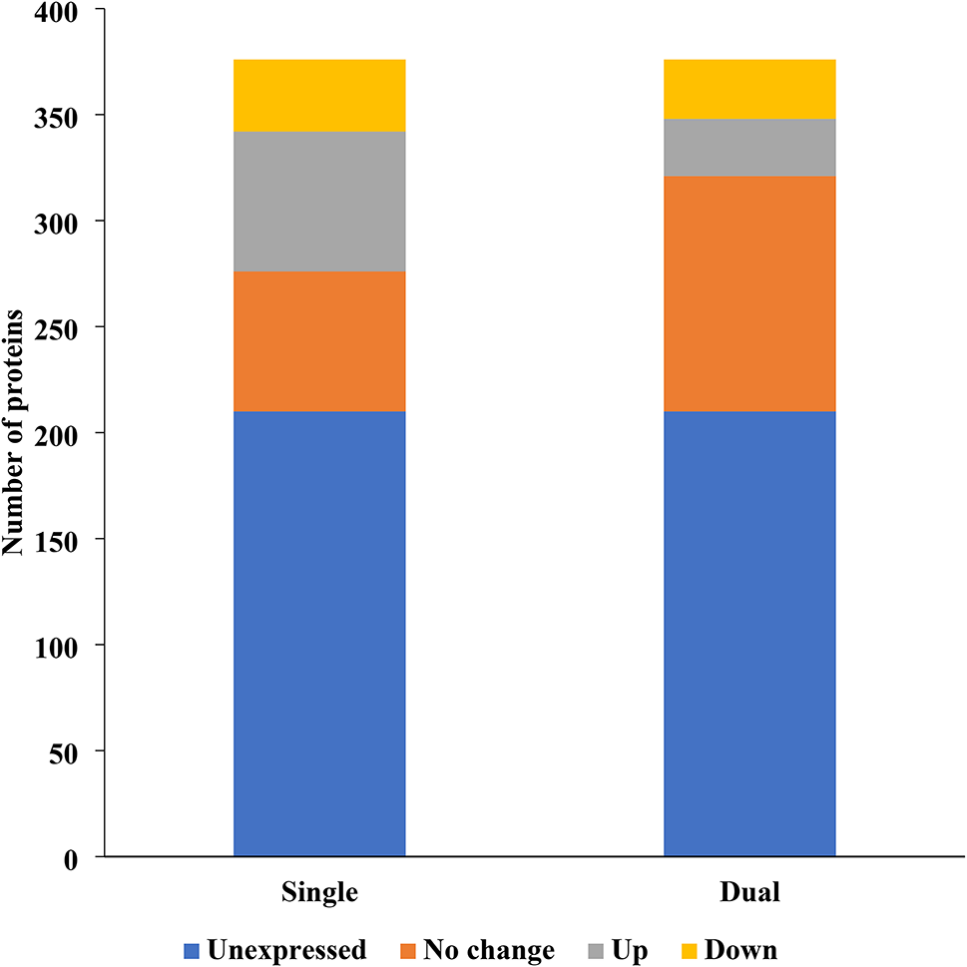
Differentially expressed proteins associated with virulence in *E. coli* CFT073 biofilms (single- vs. dual-species).

Analysis of virulence-associated proteins in *E. coli* biofilms revealed a striking effect of cohabitation with *E. faecalis*. Compared to single-species biofilms, the presence of *E. faecalis* in dual-species biofilms significantly reduced the number of overexpressed virulence proteins (Fig. 7, Table S2). Out of a total of 376 virulence proteins, the number of upregulated virulence proteins decreased from 66 in single-species biofilms to only 27 in dual-species biofilms. Interestingly, the downregulation of virulence proteins remained relatively stable, with only a slight decrease from 34 proteins in single-species biofilms to 28 in dual-species biofilms.

The analysis of differentially expressed proteins associated with virulence in single-species *E. coli* CFT073 biofilms revealed several key findings (Table S2). One prominent observation was the upregulation of proteins involved in adherence, particularly fimbriae-related proteins such as FimA, FimC, FimH, PapC, PapD, and PapH. In addition, there was significant upregulation in the cell motility category, with key flagellar components like FliC, FliG, and FliM showing increased expression. Proteins involved in immune modulation and metabolic processes, such as those involved in lipopolysaccharide biosynthesis (LpxB, RfaG), were also upregulated. However, some proteins linked to antimicrobial activity and immune modulation, including AcrB and RfaC, were downregulated. Exotoxin-related proteins, such as HlyA and ClbF, were also downregulated. Furthermore, nutritional and metabolic factors, particularly those related to iron uptake, were significantly downregulated.

In dual-species *E. coli* CFT073 biofilms with *E. faecalis*, notable shifts in protein expression were observed, particularly in adherence, motility, and immune modulation (Table S2). In the adherence category, type 1 fimbriae components such as FimH, FimF, and FimG were downregulated, while fimbrial proteins like PapH and PapE were upregulated, suggesting a complex balance between upregulated and downregulated adhesion factors. Motility-related proteins exhibited considerable variation, with flagellar components such as FliA, FlgK, and FliM being downregulated. However, many other motility proteins, including FlgF, FlgG, and FliH, were highly upregulated, indicating that motility may still play a significant role under certain conditions in dual-species biofilms. In terms of immune modulation, proteins associated with lipopolysaccharide biosynthesis, such as WecC and LpxC, were downregulated, while LpxB and AcpP were upregulated. Exotoxin production showed mixed expression, with RTX toxin hemolysin (HlyA) and colibactin components downregulated, while ClbH and ClbP were upregulated. Nutritional and metabolic factors, particularly those related to iron uptake and metabolism, were predominantly downregulated, including proteins like EntE, FepA, and HutX.

#### Differentially expressed proteins associated with virulence in *E. faecalis* ATCC 29212 biofilms

**Fig. 8:**
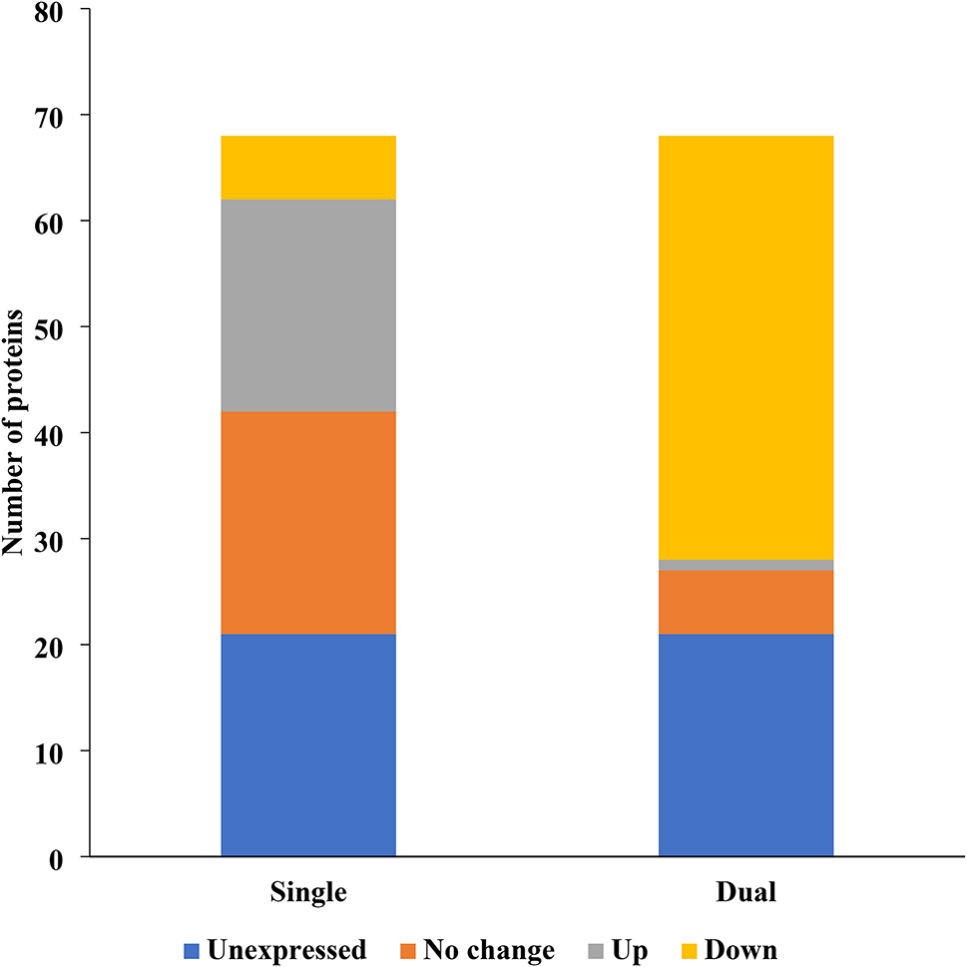
Differentially expressed proteins associated with virulence in *E. faecalis* ATCC 29212 biofilms (single- vs. dual-species).

When *E. coli* joined *E. faecalis* in biofilms (dual-species), a dramatic shift occurred in *E. faecalis*’ virulence profile. Out of a total of 68 virulence proteins, single-species *E. faecalis *biofilms exhibited a notable upregulation of virulence proteins^20^, but this number plummeted to just one protein in dual-species biofilms containing *E. coli* (Fig. 8, Table S2). Further emphasizing this suppressive influence, the number of downregulated virulence proteins in *E. faecalis* significantly increased from six in single-species biofilms to a staggering 40 in dual-species biofilms.

The analysis of differentially expressed proteins associated with virulence in single-species *E. faecalis* 29212 biofilms revealed several notable patterns (Table S2). Key findings included the upregulation of adherence-related proteins such as aldehyde dehydrogenase (Adh) and the alkaline phosphatase synthesis transcriptional regulatory protein (PhoP). Significant upregulation was also observed in biofilm-associated proteins, particularly the helix-turn-helix family protein (MalR). Exotoxin production was marked by the upregulation of several lantibiotic biosynthesis proteins and subtilase family proteins. In contrast, immune modulation proteins exhibited both up- and downregulation; while key immune modulators such as 6-phosphogluconate dehydrogenase were downregulated, glycosyltransferase family proteins and UDP-N-acetylenolpyruvoylglucosamine reductase were upregulated. Within the metabolic category, some factors like ureidoglycolate dehydrogenase (AllD) were downregulated, whereas others, particularly transport proteins, were upregulated. Stress survival proteins showed a mixed response, with superoxide dismutase (SodA) downregulated and ATP-binding protein (ClpC) upregulated.

In contrast, the analysis of differentially expressed proteins associated with virulence in dual-species *E. faecalis* 29212 biofilms with *E. coli* CFT073 revealed substantial downregulation across key functional categories (Table S2). Adherence-related proteins, such as alkaline phosphatase synthesis transcriptional regulatory protein (PhoP) and manganese ABC transporter substrate-binding lipoprotein (MntA), were significantly downregulated, showing a reduction in adhesion capacity within the dual-species biofilms. Biofilm-related proteins, including ribosylhomocysteinase (LuxS) and 2,3,4,5-tetrahydropyridine-2,6-dicarboxylate N-acetyltransferase (DapH), were also downregulated. Exotoxin production was notably reduced, with proteins involved in lantibiotic biosynthesis and the putative exotoxin CylI showing decreased expression. Immune modulation proteins, including UDP-glucose 4-epimerase (GalE) and UDP-N-acetylglucosamine 2-epimerase (MnaA), also demonstrated significant downregulation. Nutritional and metabolic factors, particularly those related to nutrient acquisition, such as ureidoglycolate dehydrogenase (AllD), phosphate ABC transporter (PstB1), and magnesium-translocating P-type ATPase (MgtA), were markedly downregulated as well. Stress survival proteins, including superoxide dismutase and various chaperonins, also exhibited consistent downregulation. The only notable exception was the upregulation of subtilase family proteins, which were significantly increased in the dual-species biofilms.

#### Differentially expressed proteins associated with antibiotic resistance in *E. coli* CFT073 and *E. faecalis* ATCC 29212 biofilms

Our analysis of the proteome data revealed intriguing patterns in the expression of antibiotic resistance proteins (Table S3). In single-species biofilms of *E. coli*, we observed upregulation of six antibiotic resistance proteins, while nine resistance proteins were surprisingly downregulated. However, in dual-species biofilms with *E. coli*, the number of upregulated resistance proteins remained consistent at six, but the number of downregulated proteins decreased to four. In contrast, *E. faecalis* exhibited a different pattern. In single-species biofilms, only three resistance proteins were upregulated, and one was downregulated. However, in dual-species biofilms, the number of upregulated resistance proteins decreased to one, while the number of downregulated proteins dramatically increased to 10. Interestingly, most differentially expressed resistance proteins in both *E. coli* and *E. faecalis *belonged to the efflux pump family. Efflux pumps represent a prevalent strategy that bacteria uses to extrude antimicrobial agents^20,21^. The prominence of these mechanisms implies that they could be a crucial tactic for both species in resisting the effects of silver, which is used as a coating on urinary catheters.

## Discussion

This study investigated the interaction between *E. coli* CFT073 and *E. faecalis* ATCC 29212 in biofilms formed on antibacterial urinary catheter segments using a dual-species culture model. Our findings revealed a complex interplay between these two uropathogens, affecting their biofilm formation and population dynamics. *E. coli* thrived initially in the presence of *E. faecalis*, reaching higher numbers than in single-species biofilms (Fig. 1A). However, this growth peaked at 48 h, followed by a decline by 72 h. This dynamic suggests a time-dependent shift in the interactions between *E. coli* and *E. faecalis*, where *E. coli* benefits early in biofilm formation but faces growth limitations as the biofilm matures. These findings are consistent with other published reports that have explored interactions between *E. coli* and *E. faecalis *in cocultures. For example, Laganenka and Sourjik^22^ observed that while *E. coli* initially overgrew *E. faecalis*, constituting the majority of the biofilm biomass at early time points. This supports our observation of *E. coli* initially dominating the biofilm in the early stages, with a peak in biomass around 48 h. Further support for our findings comes from Keogh et al.^23^, who demonstrated that *E. faecalis* can significantly augment *E. coli* biofilm growth and survival, both in vitro and in vivo. The ability of *E. faecalis* to enhance *E. coli* growth during early biofilm development may explain the initial increase in *E. coli* cell numbers that we observed. However, as the biofilm matured, the interactions between the two species likely shifted, resulting in a decline in *E. coli* numbers by 72 h. Our findings are further supported by the work of Kuznetsova et al.^24^, who identified *E. faecalis* as a key factor in promoting *E. coli* biofilm formation. They demonstrated that *E. faecalis* exports L-ornithine, which facilitates the biosynthesis of *E. coli* enterobactin siderophores, enabling *E. coli* growth and biofilm formation. This mechanism may have played a role in the early proliferation of *E. coli* observed in our study.

The dual-species biofilms encompassed a diverse range of compositions, with some biofilms predominantly composed of *E. coli*, others primarily of *E. faecalis*, and the rest showing a balanced ratio of both species (Fig. 3). This variability highlights the dynamic nature of dual-species biofilm formation and suggests that the interactions between *E. coli* and *E. faecalis* can lead to different structural outcomes depending on the local environment and competitive dynamics. This finding contrasts with a previous study in which *E. coli* and *E. faecalis *biofilms grown on silicone coupons in artificial urine medium for 24 h displayed a well-mixed composition with minimal variation^25^.

Furthermore, we observed the presence of elongated *E. faecalis* cells within the dual-species biofilms. This finding suggests possible morphological adaptations in response to the mixed-species environment (Fig. 4C). Notably, the elongated cells observed in our study are a novel discovery in the context of mixed-species biofilm cultures. Previous studies have reported the formation of elongated *E. faecalis *cells when treated with substances having antibacterial functions, such as celastrol, lupinifolin, and myristic and palmitic acids^26–28^. The presence of elongated *E. faecalis* cells in the mixed-species biofilm cultures raises intriguing questions about their role and significance. These elongated forms may contribute to the structural integrity of the biofilms, potentially enhancing their stability and cohesion. Alternatively, these elongated cells may reflect a physiological response to the interspecies interactions taking place within the biofilm.

This study pioneers the investigation of proteomic adaptations of bacteria residing within dual-species biofilms formed on antibacterial urinary catheters. By comparing comprehensive protein expression of *E. coli* and *E. faecalis* during biofilm formation, we revealed distinct strategies each bacterium employed in this complex environment. Our study showed a dynamic response by *E. coli* to biofilm formation, with a significant upregulation of proteins across various categories. This contrasts with findings by Magalhães et al., who observed a relatively low number of differentially expressed genes in *P. aeruginosa* biofilms co-cultured with *S. aureus*^29^. *E. coli*’s pronounced response suggests a more active strategy for regulating protein expression during biofilm development. This might be due to *E. coli*’s need to establish and maintain a foothold within the biofilm structure compared to *P. aeruginosa*, which potentially exhibits a more dominant or pre-adapted biofilm formation strategy.

In *E. coli* biofilms, the Cell motility (N) category showed a significant upregulation, suggesting an emphasis on movement within the biofilms (Fig. 5C). Consistent with this observation, proteins associated with the motility complex were found to be overexpressed during biofilm formation of *Campylobacter jejuni* and *Rhizobium etli*^30,31^. Similarly, studies suggest that flagellum-mediated motility might be important for *Listeria monocytogenes *biofilm development by facilitating recruitment of motile cells^32^. However, the presence of *E. faecalis* in dual-species biofilms resulted in a considerable reduction in the upregulation level of this category. This indicates that *E. coli* adjusts its motility in response to the presence of *E. faecalis*. Interestingly, this finding contrasts with a previous study, which reported no significant difference in the abundance of motility-associated proteins in mixed biofilms of *Porphyromonas gingivalis* and *Treponema denticola *compared to planktonic cells^33^. Also interesting was a downregulation of proteins associated with Cell wall/membrane/envelope biogenesis (M) in both single- and dual-species biofilm types (Fig. 5C), suggesting that *E. coli* may focus on establishing motility and biofilm formation before investing heavily in cell wall maintenance. Across single- and dual-species biofilm types, *E. coli* exhibited high levels of protein production (Translation, ribosomal structure and biogenesis - J), indicating a continuous need for protein synthesis during biofilm development. The regulation of gene expression (Transcription - K) appeared balanced, suggesting a fine-tuned control over various cellular processes.

*E. faecalis* displayed a modest increase in upregulated proteins compared to downregulated proteins when grown as a single-species biofilm. However, this trend dramatically reversed in dual-species biofilms with *E. coli*, with a significant reduction in upregulated proteins and a substantial increase in downregulated proteins. This observation aligns with previous studies in which *P. aeruginosa* grown in dual-species cultures with *S. aureus *downregulated genes associated with metabolism and cellular processes^29,34^.

These findings suggest that *E. faecalis* undergoes a significant metabolic adaptation in response to the presence of *E. coli*, potentially involving a decrease in overall activity. This is further supported by Verhaegh et al.’s work, which demonstrated that genes involved in protein biosynthesis, transport, signal transduction, and energy metabolism were downregulated in mixed biofilms of *Streptococcus pyogenes* and *Moraxella catarrhalis*^35^. *E. faecalis* adopted a more balanced approach to protein regulation in single-species biofilms. The Cell wall/membrane/envelope biogenesis (M) category showed upregulation (Fig. 6C), implying a focus on constructing a robust cell wall for biofilm development. However, the presence of *E. coli* in dual-species biofilms resulted in a dramatic shift in *E. faecalis*’ protein regulation. Most COG categories, including Translation (J), Transcription (K), and those related to transport processes (E, F, G), exhibited significant downregulation (Fig. 6C). This suggests a substantial response to cohabitation, potentially involving a decrease in overall activity and a metabolic adaptation.

The differential expression of virulence-associated proteins in *E. coli* and *E. faecalis* biofilms provides intriguing insights into their adaptive strategies in single- and dual-species environments. In *E. coli* biofilms, the presence of *E. faecalis* significantly reduced the number of overexpressed virulence proteins, suggesting a notable effect of cohabitation. *E. faecalis* displayed a drastic shift in its virulence profile when cohabiting with *E. coli*. The number of upregulated virulence proteins dramatically decreased, while the number of downregulated proteins significantly increased. This aligns partially with findings reported by Tognon et al., who observed repression of virulence factors, including capsule biosynthesis genes, in *S. aureus* biofilms during co-culture with *P. aeruginosa*^36^. However, contrasting observations have been reported by Miller et al., who found a more complex response in *S. aureus* biofilms co-cultured with *P. aeruginosa*. In their study, secretion and capsule biosynthesis virulence factors were upregulated, while adhesion and invasion factors were downregulated^34^. Additionally, in a polymicrobial wound infection model, the virulence factors Panton-Valentine leukocidin and α-hemolysin of *S. aureus* were overexpressed when grown with *P. aeruginosa*^37^.

Furthermore, several virulence markers associated with cytotoxicity, stress response, quorum sensing, and biofilm formation were found to be upregulated in dual-species biofilm cultures of *S. aureus* with *P. aeruginosa*^38^. These contrasting observations highlight the complex and context-dependent changes in the expression of virulence factors that can result from bacterial species interactions in biofilms. The specific outcomes may vary depending on the microbial species and the environmental conditions, emphasizing the need for further research to fully understand these intricate interactions.

While this study sheds light on the dynamic interactions between *E. coli* and *E. faecalis* during biofilm formation on catheters, several aspects call for further exploration. The current investigation focused on a single time point (72 h) for whole protein sequencing. Although informative, incorporating additional time points throughout biofilm development could provide a more complete picture of how protein expression evolves during biofilm maturation. Additionally, the study primarily investigated biofilms formed by *E. coli* and *E. faecalis*. However, urinary catheters are susceptible to colonization by a wider range of bacteria. Future studies could explore protein expression dynamics and biofilm formation in multi-species communities, reflecting the natural complexity of catheter-associated infections.

Although artificial urine media offers a controlled environment for experimentation, it may not fully replicate the intricate environment of the human urinary tract. The urinary tract is a dynamic system with various factors influencing biofilm formation and protein expression. To bridge this gap, future research could utilize animal models of urinary tract infections. This would enable a more comprehensive understanding of protein expression and biofilm development in a more realistic setting.

While our analysis focused on whole proteome profiles, we acknowledge the inherent heterogeneity of dual-species biofilms, which can impact the interpretation of proteome data. The observed variations in the balance between *E. coli* and *E. faecalis* within these biofilms highlight the importance of considering spatial factors and the potential for distinct subpopulations with unique proteomic signatures. Future studies should incorporate spatial analysis techniques to examine the distribution of *E. coli* and *E. faecalis* within individual biofilms. By correlating spatial information with proteome profiles, we can gain a more nuanced understanding of the factors driving biofilm composition and function. Furthermore, the development of advanced proteomic techniques that can analyze small groups of cells within biofilms would enable a more detailed investigation of the heterogeneity at the cellular level. Such approaches could uncover distinct subpopulations with specialized functions within the biofilm community.

Our proteome analysis was conducted at a single time point and therefore did not capture the dynamic changes in protein expression that occur as the biofilm matures over time. This limitation restricts our ability to fully understand the temporal aspects of biofilm development and the evolving interactions between *E. coli* and *E. faecalis*. Future studies should aim to perform proteome analyses at multiple time points to provide a comprehensive view of the temporal dynamics in dual-species biofilms.

## Conclusion

This study explores the intricate interplay between *E. coli* and *E. faecalis* in urinary catheter biofilm formation using a dual-species culture model. Results revealed dynamic changes, with initially thriving *E. coli* populations declining during biofilm development, while *E. faecalis* exhibited lower initial growth in dual-species biofilms. The composition of biofilms varied, with some being predominantly *E. coli*, others primarily *E. faecalis*, and some displaying a balanced ratio. Global proteomic analysis uncovered distinct adaptive strategies, with *E. coli* emphasizing motility, transcription, and protein synthesis, and *E. faecalis* exhibiting a conservative approach. Both species downregulated virulence-associated proteins in dual-species biofilms.

## Electronic supplementary material

Below is the link to the electronic supplementary material.

Supplementary Material 1

Supplementary Material 2

Supplementary Material 3

## Data Availability

Data is provided within the manuscript or supplementary information files.
